# Role of positron emission tomography-computed tomography in endometrial cancer

**DOI:** 10.4274/tjod.24572

**Published:** 2017-12-30

**Authors:** Evrim Erdemoğlu, Sevim Süreyya Çerçi, Ebru Erdemoğlu, Yakup Yalçın, Burak Tatar

**Affiliations:** 1 Süleyman Demirel University Faculty of Medicine, Department of Gynecologic Oncology, Isparta, Turkey; 2 Süleyman Demirel University Faculty of Medicine, Department of Nuclear Medicine, Isparta, Turkey; 3 Maternity Hospital, Clinic of Obstetrics and Gynecology, Isparta, Turkey

**Keywords:** Endometrial cancer, maximum standardized uptake value, positron emission tomography, diagnosis, prognostic factors

## Abstract

**Objective:**

The efficacy of preoperative ^18^F-fluoro-D-glucose (^18^F-FDG) positron emission tomography-computed tomography (PET-CT) in endometrium cancer is controversial. We examined the efficacy of PET-CT and the association between maximum standardized uptake value (SUVmax) and prognostic factors in endometrial cancer.

**Materials and Methods:**

Thirty patients with endometrial cancer underwent preoperative ^18^F-FDG/PET-CT. The patients were treated with abdominal hysterectomy with bilateral salpingo-oophorectomy, and bilateral systemic pelvic lymphadenectomy was planned for all patients; paraaortic lymphadenectomy was performed in patients with intermediate and high risk. Tumor histology, grade, depth of myometrial invasion, maximum tumor diameter, lymphovascular invasion, nodal status, and ovarian/adnexal metastases were recorded.

**Results:**

The mean primary tumor diameter was reported smaller in PET-CT and the effect size of PET-CT was -0.60. The kappa value was 0.06 for myometrial invasion. Pelvic lymph node metastasis was reported in 22.2% of patients in PET-CT. However, 3.7% of patients had pelvic lymph node metastasis. The kappa value for pelvic lymph node metastasis was 0.23, and sensitivity, specificity, and positive and negative predictive values were 100%, 80.7%, 16.6%, and 100%, respectively. Paraaortic lymph node metastasis in PET-CT was suspected in 10%. However, paraaortic lymph node metastasis was found in 6.7% in histopathologic analyses. The kappa value was 0.15. The sensitivity, specificity, and positive and negative predictive values of PET-CT for detecting paraaortic lymph node metastases were 100%, 93.7%, 66.6%, and 100%, respectively. Myometrial invasion and tumor diameter were the only important prognostic factors affecting SUV_max._

**Conclusion:**

According to our results, PET-CT has a limited role and diagnostic efficacy in endometrial cancer. The indications of FDG/PET-CT in endometrium cancer should be studied further and revised.

## PRECIS:

Positron emission tomography-computed tomography has a limited role and diagnostic efficacy in endometrial cancer.

## INTRODUCTION

Endometrial cancer is the most common gynecologic cancer; the estimated new cases and deaths in 2015 were 54.870 and 10.170, respectively^(1)^. The majority of cases are confined to the uterus and diagnosed at an early stage^(1)^. Endometrium cancer spreads primarily by direct extension, lymphatic channels, trans tubal migration, and blood vessels. The main lymphatic metastases involve pelvic lymph nodes, and less commonly presacral, paraaortic, and inguinal lymph nodes^(2)^. Until 1988, endometrium cancer was staged clinically^(3)^. After finding evidence that clinical staging was inaccurate, the International Federation of Gynaecology and Obstetrics (FIGO) staging of endometrium cancer changed to a surgical-pathologic-based system. Surgical staging of endometrium cancer provides prognostic factors that are not available before surgery, or which are not always in exact concordance before and after surgery. Prognostic factors are the stage, tumor histology, grade, depth of myometrial invasion, lymphovascular space invasion (LVSI), tumor diameter, extrauterine spread, and lymph node metastasis^(2,3)^.

Positron emission tomography (PET) uses the detection of enhanced glucose metabolism in malignant tumors based on the uptake of ^18^F-fluoro-D-glucose (^18^F-FDG) for functional diagnosis of malignant tumors. PET-computed tomography (CT) allows simultaneous imaging of anatomic and metabolic information. ^18^F-FDG/PET-CT has been widely used in clinical practice for tumor detection, staging, treatment monitoring and detection of disease recurrence. Preoperative PET-CT imaging is not universally accepted in endometrial cancer, and the efficacy and accuracy of ^18^F-FDG/PET-CT in endometrium cancer is controversial and may be overestimated. In this study, we examined the efficacy of PET-CT in a non-stratified patient group with endometrium cancer in a tertiary university setting over a 1-year period. We also analyzed the association between maximum standardized uptake values (SUV_max_) and prognostic factors based on final pathology.

## MATERIALS AND METHODS

### Subjects

Patients diagnosed as having endometrial cancer histopathologically who underwent PET-CT for treatment planning in our department between 2014 and 2015 were included in the study. Patients who did not undergo surgery or preoperative PET-CT imaging were not recruited. Patients who had neoadjuvant treatment before the operation, claustrophobia, and uncontrolled diabetes mellitus (random blood sugar >200 mg/dL) were excluded. The standard preoperative procedures included clinical examination, chest X-ray, and abdominal and vaginal ultrasonography. Laboratory examinations including routine hematology, biochemistry, cancer antigen-125 (CA-125) were performed. Routine preoperative imaging for myometrial invasion was not performed because it was not found to be cost-effective. The study was approved by Süleyman Demirel University Ethics Committee (approval number: 54).

### Surgery

All patients had a midline laparotomy and abdominal hysterectomy with bilateral salpingo-oophorectomy under general anesthesia. Panniculectomy was performed in two patients for staging purposes. Bilateral systemic pelvic lymphadenectomy was planned for all patients; paraaortic lymphadenectomy was performed in patients with intermediate and high risk. Lymph node dissection was performed en-bloc without dividing for separate regions for oncologic safety. Suspicious nodes on FDG/PET-CT were marked. Analysis of matching for the PET-CT and histopathology was made for the whole nodal chain including the marked lymph node(s).

### Positron emission tomography-computed tomography protocol and image analysis

Whole-body ^18^F-FDG/PET-CT images were performed using a PET/CT scanner (Philips Gemini TF), consisting of dedicated lutetium yttrium oxyorthosilicate full-ring PET scanner and 64-slice CT. All patients were kept fasting for six hours before intravenous (i.v.) injection of 3.7 MBq/kg (0.1 mCi/kg) of ^18^F-FDG. During the 60 min. waiting period, all patients were orally hydrated with around 1.5 L of contrast. After 60 min., the combined examination was started by asking the patients to empty their bladder and injecting the i.v. contrast. The CT scan was acquired first, followed by the PET scan. PET and CT images (non-corrected and attenuation-corrected) were evaluated in the rotating maximum-intensity projection and in the cross-sectional planes view (transverse-sagittal-coronal). The ^18^F-FDG uptake in the primary tumor was quantified using SUV_max_ measurements.

### Pathological evaluation

Tumor histology, grade, depth of myometrial invasion, maximum tumor diameter, lymphovascular invasion, nodal status, ovarian/adnexal metastases, and cytology were recorded. Each primary tumor and dissected lymph nodes were sliced and stained with hematoxylin and eosin (H&E) and examined microscopically by at least one gynecologic pathologist. Pathologist(s) were blinded to the PET/CT results. Discordant results on lymph nodes were further evaluated by ultra-staging. Ultra-staging involved cutting an additional two adjacent 5-μm sections at 2 levels 50-μm apart from each paraffin block that lacked metastatic carcinoma on routine H&E staining. At each level, one slide was stained with H&E and with immunohistochemistry using anti-cytokeratin.

### Prognostic factors

Prognostic factors evaluated were tumor histology, grade, depth of myometrial invasion, maximum tumor diameter, lymphovascular invasion, nodal status, elevated CA-125, and thrombocytosis. Grade 1 and 2 tumors were classified as low grade; grade 3 tumors were classified as high grade.

### Statistical Analysis

Statistical analyses were performed using MedCalc for Windows, version 12.5 (MedCalc Software, Ostend, Belgium) and p values less than 0.05 were considered statistically significant.

**Analyses of consistency and agreement between positron emission tomography-computed tomography and histopathology: Continuous variables**

The mean and standard derivation of continuous variables were calculated using the t-test. The importance of the difference between means was evaluated through effect size. Effect size measures how much the mean in PET-CT exceeded the mean of the histopathologic measurement. Effect size <0.20 was considered small; 0.50 was moderate effect size, and >0.80 was large effect size. The consistency and agreement of the continuous variables (tumor diameter) were evaluated using the intraclass coefficient and concordance correlation coefficient. Concordance correlation coefficient <90 indicates poor correlation and agreement. Intraclass correlation coefficient (ICC) was analyzed for absolute agreement and consistency. ICC were calculated for each single measurement and also for average (mean) values; ICC >70 indicated poor agreement, >80 indicated moderate agreement, >90 good agreement, and 100 meant perfect agreement.

### Analyses of consistency and agreement between positron emission tomography-computed tomography and histopathology: Ordinal and nominal variables

The frequency of variables was calculated, and kappa statistics was used to analyze agreement between PET-CT and histopathologic findings. Kappa value is 1 when there is perfect agreement between PET-CT findings and histopathologic findings. Kappa value is zero when there is no agreement better than chance. Kappa value is negative when the agreement is worse than chance. Kappa index (inter-rater agreement concordance, in ordinal or nominal scales) is usually interpreted according to qualifiers as “poor” (<0.20), “slight” (0.20-0.40), “fair” (0.41-0.60), “moderate” (0.61-0.80), and 0.81-1 as almost perfect agreement.

### Analyzes of maximum standardized uptake value and high-risk prognostic indicators

Median and interquartile ranges SUV_max_ for each prognostic indicator such as histology, grade, myometrial invasion, lymphovascular invasion, and lymph node metastasis were calculated. The Mann-Whitney U test or Kruskal-Wallis test was used to analyze primary tumor SUV_max_ and prognostic indicators. Regression analyses were used to study the relation between tumor diameter in pathologic measurement and primary tumor SUV_max_.

## RESULTS

A total of 30 patients were included in the study. The mean age of patients was 58.8±9.3 years. Eighty-six percent of the women were postmenopausal. Endometroid histology was reported in 90% of patients. Serous histology was reported in 10% of patients. Tumor was grade 1, 2, and 3 in 43.3%, 40%, and 16.7%, respectively. LVSI was observed in 6.7% of cases. Pelvic lymphadenectomy was planned for all patients. However, the procedure was abandoned during the operation in two patients because of co-morbidities and was not technically feasible and safe in one patient. Pelvic and paraaortic lymphadenectomy were performed in 27/30 and 18/30 of patients, respectively. The mean number of pelvic lymph nodes harvested was 18.6±10.6. The mean number of harvested paraaortic lymph nodes was 10.5±5.4. The mean blood glucose level was 110±23.1 mg/dL. The mean CA-125 level was 25.5±39.1 IU/mL. Thirteen percent of patients had CA-125 higher than the reference value of 35 IU/mL. Seven percent of patients had thrombocytosis. There was no positive cytology.

### Agreement between positron emission tomography-computed tomography and pathologic findings

**Figure 1:**
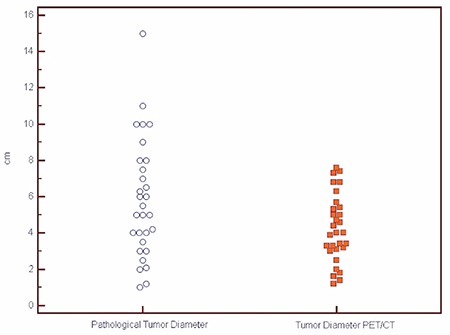
Tumor size estimate in positron emission tomography-computed tomography and histopathologic measurement PET/CT: Positron emission tomography/Computed tomography

The mean primary tumor diameter was reported as 4.2±1.8 cm in PET-CT. The man primary tumor diameter was measured as 5.8±3.2 cm in the specimen. The effect size was found to be -0.60, indicating a modest change in diameter of tumor in PET-CT and histopathology. Tumor diameter was poorly correlated with measurements taken in PET-CT. Consistency and absolute agreement of PET-CT and histopathologic analyses are shown in Table 1 and Figure 1.

Thirty-three percent had myometrial invasion in PET-CT; however, 93.3% had myometrial invasion in the specimen. A kappa value of 0.06 [95% confidence interval (CI): -0.0303-0.168] was found for myometrial invasion in PET-CT and histopathology.

Pelvic lymph node metastasis was reported in 22.2% in the PET-CT reports. However, 3.7% of patients had pelvic lymph node metastasis. The kappa value for pelvic lymph node metastasis was 0.23 (95% CI: -0.15-0.62). There was fair consistency between PET-CT and histopathologic findings, and the sensitivity, specificity, and positive and negative predictive values were 100%, 80.7%, 16.6%, and 100%, respectively. Paraaortic lymph node metastasis in PET-CT was suspected in 10%. However, paraaortic lymph node metastasis was found in 6.7% in histopathologic analyses. The kappa value was 0.15 (95% CI: -0.0749-0.387). There was no agreement between PET-CT and histopathologic findings. The sensitivity, specificity, and positive and negative predictive values of PET-CT for detecting paraaortic lymph node metastases were 100%, 93.7%, 66.6%, and 100%, respectively.

### Factors effecting primary tumor maximum standardized uptake value

**Figure 2:**
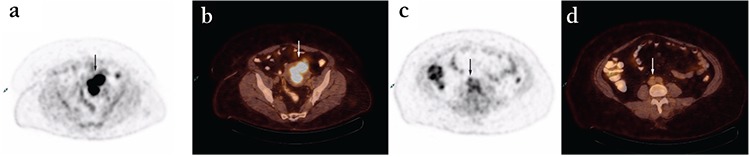
A 60-year-old female with endometrial cancer. Fluoro-D-glucose/positron emission tomography-computed tomography images demonstrate increased fluoro-D-glucose uptake in the tumor (a,b) and metastatic inter aortocaval lymph node (c,d)

Myometrial invasion and tumor diameter were the only significant factors affecting SUV_max_ (Tables 2, 3). The median SUV_max_ of tumors with no myometrial invasion was 5.2. However, the median SUV_max_ of tumors with myometrial invasion less than 50% and more than 50% were 10.6 and 18.4, respectively (Figure 2). Primary SUV_max_ was not statistically significantly higher in high grade, LVSI positive, and lymph node positive patients. Tumor size and SUV_max_ were correlated; as the tumor size enlarged, SUV_max_ values increased (Table 3). SUV_max_ was not affected by high CA-125 values or thrombocytosis.

## DISCUSSION

Pre-operative imaging in endometrium cancer may help to plan surgery by adding or omitting staging procedures. Secondly, imaging may help to identify candidates for fertility-sparing treatment. Another indication of pre-operative imaging could be in patients who cannot undergo surgery because of co-morbidities^(4)^. We found the efficacy of ^18^F-FDG/PET-CT was low in endometrium cancer, and ^18^F-FDG/PET-CT was not in concordance with histopathologic findings. We also analyzed prognostic factors that affected SUV_max_ values. The number of studies in this regard is few and the results conflicting(5,6); most detected only lymph node metastasis, rather than other clinicopathologic prognostic factors.

Women with endometrium cancer are stratified into risk groups for recurrence and treatment planning. The French Multicenter Collaboration study and others identified that tumor size larger than 2-3 cm was a poor prognostic factor^(7, 8, 9)^. Tumor size is correlated with myometrial invasion, nodal metastases, peritoneal cytology, CA-125 levels, advanced disease, and relapse^(8,9)^. The size of the primary tumor was moderately consistent with histopathologic tumor measurement, and tumor diameter was predicted smaller in PET-CT scans in our study. Myometrial invasion is a part of staging in endometrium cancer, and PET-CT was not useful in predicting myometrial invasion; 33% of patients had myometrial invasion in PET-CT, whereas 93.3% had myometrial invasion in the specimen. The sensitivity and specificity of CT in detecting myometrial invasion range from 40% to 83% and 42% to 75%, respectively^(10)^. Sudo et al.^(11)^ reported that patients with endometrium cancer should first be triaged by myometrial invasion in magnetic resonance imaging (MRI) before PET-CT. Their report on 37 patients with endometrium cancer showed that PET-CT could identify risk groups after MRI triage. FDG/PET-CT has limitations in the evaluation of the depth of myometrial invasion and in defining tumor borders^(12)^.

Lymphatic metastases are one of the most important prognostic factors in endometrium cancer. There are various imaging modalities to assess for lymph node metastasis before surgery. Park et al.^(13)^ compared PET-CT and MRI to detect lymph node metastases in patients with endometrial cancer, and reported that the sensitivity and specificity of FDG/PET-CT was better than MRI for detecting metastatic lymph nodes in patients with endometrial cancer. Pelvic and paraaortic lymph node metastases were over-estimated by FDG/PET-CT in our study. We found fair consistency for pelvic lymph nodes and no consistency for paraaortic lymph nodes. Our findings for detecting lymph node metastases using FDG/PET-CT are in agreement with other studies. Gholkar et al.^(14)^ reported that FDG/PET-CT had a sensitivity of 100%, specificity of 61.11%, positive predictive value of 22.22%, negative predictive value of 100%, and accuracy of 65% for pelvic lymph node metastases; sensitivity of 100%, specificity of 66.67%, positive predictive value of 20%, negative predictive value of 100%, and accuracy of 69.23%^(15)^.The high sensitivity and negative predictive value of FDG/PET-CT are important in the detection of patients who are truly lymph node-negative^(15)^. However, FDG/PET-CT has a high false positive rate for lymph nodes. Besides, FDG/PET-CT cannot detect metastatic lymph nodes smaller than 5 mm^(15)^.

Analyses of prognostic factors and SUV_max_ values showed that SUV_max_ was significantly higher in patients with deeper myometrial invasion and higher tumor diameter. However, we found no correlation between SUV_max_ and tumor histology, tumor grade, lymphovascular invasion, and lymph node metastases in contrast to other reports^(16,17)^. Kitajima et al.^(18)^ reported that SUV_max_ was not a good index for preoperative stratifying patients into high risk and low risk, and suggested the use of metabolic tumor volume and total lesion glycolysis instead of SUV_max_.

### Study Limitations

The limitations of our study were similar to other reports; its design was retrospective, and a relatively small number of patients were included in a single tertiary center. Another limitation of our study may be that paraaortic lymphadenectomy was not performed in all cases due to technical considerations or due to other pathologic findings indicating low risk.

There are also diagnostic limitations of FDG/PET-CT in endometrial cancer due to the natural slow progress of the disease. The majority of patients with endometrial cancer have the endometrioid type, are early stage and low risk without lymph node metastases. The low prevalence of poor prognostic factors may cause a bias towards a lower positive predictive value and higher negative predictive value. We did not stratify patients by preoperative histopathologic findings or other imaging techniques. We rather preferred to include a non-stratified population of patients who were referred to the gynecologic oncology department in one year.

## CONCLUSION

Surgical staging and obtaining prognostic factors using the FIGO staging is the standard of treatment for endometrial cancer, although lymphadenectomy for early-stage low-risk patients is controversial. Although the pre-operative use of PET-CT in endometrium cancer is increasing, the results of our study and others cast doubt on the diagnostic efficacy of PET-CT in endometrium cancer(19). The indications of FDG/PET-CT in endometrium cancer should be revised and studied further.

## Figures and Tables

**Table 1:**
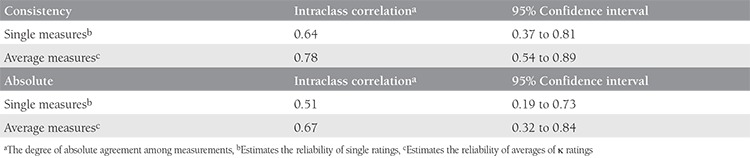
Intraclass correlation coefficient analyses for tumor diameter

**Table 2:**
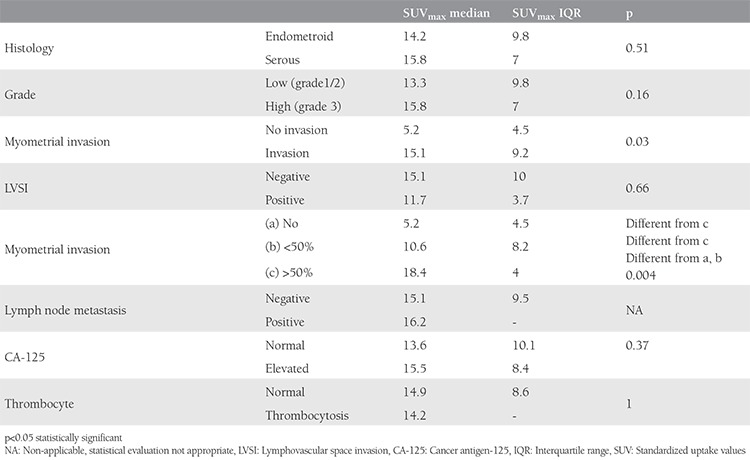
Maximum standardized uptake value and histopathologic prognostic factors

**Table 3:**
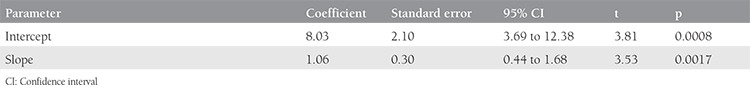
Regression analyses between primary tumor maximum standardized uptake value and tumor diameter primary tumor maximum standardized uptake value=8.03+1.06 diameter
